# A 95% decline in estimated newly acquired HIV infections, Amsterdam, 2010 to 2022

**DOI:** 10.2807/1560-7917.ES.2023.28.40.2300515

**Published:** 2023-10-05

**Authors:** The HIV Transmission Elimination AMsterdam (H-TEAM) Initiative, The HIV Transmission Elimination AMsterdam (H-TEAM) Initiative, Ard van Sighem, Sally Hendriks, Febe Deug, Paul Zantkuijl, Jan EM van Bergen, John de Wit, Janneke Heijne, Elske Hoornenborg, Tom van Benthem, Maarten F Schim van der Loeff, Maria Prins, Udi Davidovich, Suzanne E Geerlings, Dinah Bons, Michiel Heidenrijk, Pieter Brokx, Nina Schat, Peter Reiss, Marc van der Valk, Godelieve J de Bree

**Affiliations:** 1 The members of the HIV Transmission Elimination AMsterdam (H-TEAM) Initiative are listed under Investigators

**Keywords:** The Netherlands, Amsterdam, fast track city, city-approach, sexually transmitted infections, viral infections, HIV infection, infection control, public health policy, epidemiology, modelling, policy

## Abstract

The infrastructure in cities provides unique opportunities to eliminate HIV. Since 2014, the HIV Transmission Elimination AMsterdam Initiative, a consortium involved in HIV prevention and care, has employed an integrated approach to curb HIV incidence in Amsterdam. This effort contributed to the 95% decline in estimated newly acquired infections and the 79% decline in observed new HIV diagnoses in Amsterdam from 2010 to 2022. In 2022, Amsterdam reached and exceeded the 95–95–95 UNAIDS treatment cascade goals (98–95%-96%).

Worldwide, around 8 million people with HIV (PWH) live in urban areas [1], where social, economic and structural factors drive inequality in access to health services and thereby contribute to the propagation of the HIV epidemic within a city. At the same time, cities may have strong prevention and care infrastructures that could be exploited to curb the ongoing transmission of HIV [1]. The potential of interventions to contain HIV in urban areas has previously been demonstrated in several cities [2,3]. 

In 2014, *The HIV Transmission Elimination Amsterdam Initiative* (H-TEAM Initiative) [4] was founded with the aim to develop an integrated approach to curb HIV incidence in Amsterdam [5]. In the present communication, we describe this H-TEAM Initiative approach in relation to the marked decline in the HIV epidemic in Amsterdam.

## The situation in 2010 and the H-TEAM Initiative’s approach 

Key to the design and evaluation of the H-TEAM Initiative’s success is the availability of data on HIV prevalence, incidence and transmission in Amsterdam. Data on HIV diagnoses and prevalence, as part of the AIDS Therapy Evaluation in the Netherlands (ATHENA) cohort, are collected by Stichting HIV Monitoring (SHM) from over 98% of all PWH in care in the Netherlands [6]. People entering HIV care receive written information about participation in the ATHENA cohort and are informed by their consulting physician of the purpose of the data collection, after which they can consent verbally or elect to opt out. Data are pseudonymised before they are provided to researchers and may be used for scientific purposes, including calculation of HIV incidence. Data captured by SHM include viral sequence data that are used to investigate transmission chains in Amsterdam; a recent study showed that in the period from 2014 to 2018, an estimated 67% of new infections had an Amsterdam resident as source [7]. These data underscore that there is considerable potential to prevent HIV infections among Amsterdam residents through city-level interventions.

**Figure 1:**
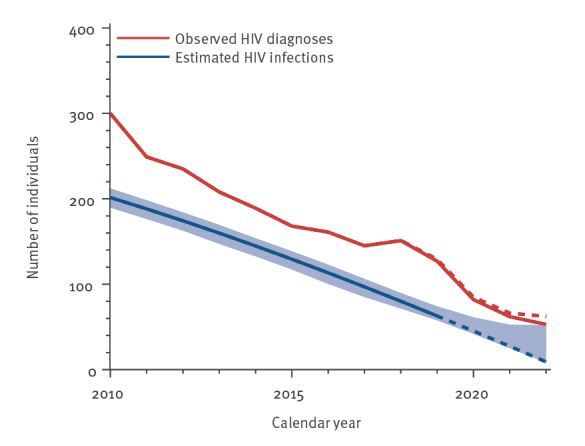
Annual number of observed HIV diagnoses and estimated newly acquired HIV infections, Amsterdam, 1 October 2013

In 2010, 4 years before the start of the H-TEAM Initiative, 300 people were newly diagnosed with HIV in Amsterdam, of whom 234 (78%) were in men who have sex with men (MSM). Based on modelling [8], the estimated number of newly acquired infections in that year was 201 (95% confidence interval (CI): 190–212) (Figure 1).

Source for the calculations: European Centre for Disease Prevention and Control HIV Modelling Platform [8].

Red dashed line: number of diagnoses after adjusting for the delay in notification to SHM; blue zone: uncertainty around the estimate; blue dashed line: estimates in 2020 and later, which are still uncertain, as these are sensitive to the observed number of diagnoses in those years.

**Figure 2:**
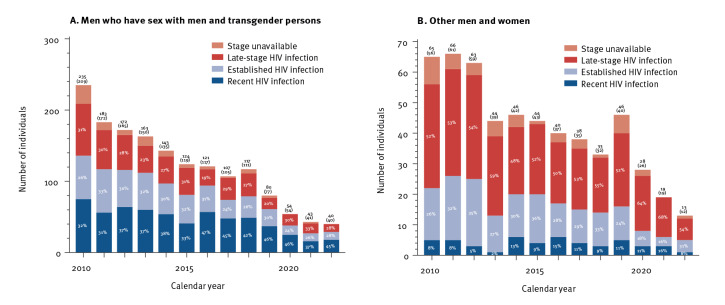
Number and proportion of individuals diagnosed with recent, established or late-stage HIV infection, Amsterdam, 1 October 2023 (n = 2,127)

At that time, several HIV test and treat strategies were in place. These included, since 2007, the introduction of opt-out testing for HIV (i.e. HIV included in STI testing unless actively declined) at the large Center for Sexual Health (CSH) of the Public Health Service Amsterdam, online test facilities for MSM (*Man tot Man Testlab*) since 2009, and the early uptake of the recommendation to initiate anti-retroviral therapy (ART) regardless of CD4^+^ T-cell counts (since 2010) and during early HIV infection (since 2012). Together, these standard prevention and HIV care measures collectively resulted in a continued sustained decline in the annual number of new HIV diagnoses and incident infections after 2010 (Figures 1 and 2) [9]. Figure 1 shows that the number of estimated new infections was smaller than the number of new diagnoses. In the case of Amsterdam, for people who acquired HIV in 2010 or later, the average time to diagnosis was approximately 3 years, with a median time of 2.2 years (interquartile range: 1.1–4.1). This means that the trend in annual numbers of newly acquired HIV infections will be reflected in annual number of new HIV diagnoses after approximately 3 years, as is the case in Figure 1 (horizontal distance between the blue and the red line). It indicates that the trend in new diagnoses and new infections reflects the dynamics of the HIV epidemic in Amsterdam. 

Recent HIV infection: (i) a negative or indeterminate blot at the time of diagnosis, or (ii) a last negative test at most 12 months before diagnosis. Established HIV infection: no recent HIV infection, CD4^+^ T-cell counts above 350 cells/mm^3^, and not having AIDS at the time of diagnosis. Late-stage HIV infection: no recent HIV infection, CD4^+^ T-cell counts below 350 cells/mm^3^ or having AIDS, regardless of CD4^+^ T-cell count.

Numbers above the bars: total diagnoses in each year; numbers in brackets: diagnoses excluding individuals whose stage at diagnosis was unavailable. Percentages inside the dark blue bars are relative to total number of diagnoses. Please note the different scales on the Y-axes.

**Figure 3:**
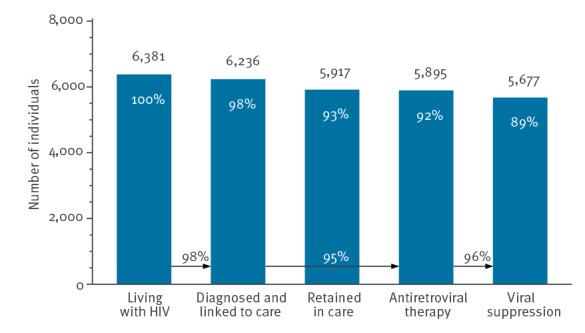
Continuum of care for the total estimated population with HIV living in Amsterdam by the end of 2022 (n = 6,375)

To achieve a substantial improvement in the decline in the number of new HIV infections, the H-TEAM Initiative brought together relevant stakeholders from public health, civil society, key affected communities including the Dutch patient association and the PrEP community group, general practitioners and HIV physicians in Amsterdam. The Initiative aimed to design and implement a multidisciplinary and integrated approach to reduce incidence of HIV infection. This approach consists of combination interventions including PrEP, innovative test-and-treat strategies at general practioners’ [10], CSH and hospitals, and research studies on motives and barriers for testing. All interventions are implemented simultaneously and each target a pillar of the HIV care continuum [5,11]. This concerted effort is likely to have contributed significantly to the 95% decline in estimated newly acquired HIV infections from 201 (95% CI: 190–212) in 2010 to nine (95% CI: 8–52) in 2022 (Figure 1), the 79% decline in observed new HIV diagnoses in Amsterdam from 300 in 2010 to 62 in 2022 (Figure 2) and Amsterdam having reached, and exceeded, the 95–95–95 UNAIDS goals in 2022 (Figure 3). In 2022, an estimated 6,375 (95% CI: 6,354–6,445) PWH were living in Amsterdam, of whom 6,236 (98%) had been diagnosed, 139 (95% CI: 117–208) individuals remained undiagnosed, and 5,895 (95%) of the diagnosed had started ART. Of those who had started ART, 5,677 (96%) had a suppressed viral load below 200 copies/mL. The continuum of care shows that 319 people were considered lost to follow-up. There are no data available on people who tested positive but never enrolled in HIV care. However, the number never enrolling in HIV care is most likely to be very small.

The percentages at the top of the bars are calculated relative to the number living with HIV, while percentages at the bottom correspond to the UNAIDS 95–95–95 targets for 2025. Individuals were considered to be retained in care if they had at least one HIV RNA or CD4^+^ T-cell count measurement or a clinic visit in 2022. Viral suppression was defined as a most recent HIV RNA measurement in 2022 below 200 copies/mL. Numbers were adjusted for the delay in notification to Stichting HIV Monitoring.

## The H-TEAM Initiative’s interventions on prevention of new infections and immediate test and treat

In 2015, HIV pre-exposure prophylaxis (PrEP) was introduced in Amsterdam for individuals who can benefit from PrEP, mainly MSM and transgender persons (TGP). The Amsterdam PrEP demonstration project (AMPrEP), which started in 2015 as part of H-TEAM, showed a high uptake by MSM and TGP, good acceptability and usability of PrEP and of the choice between daily or event-driven use [12]. These results from AMPrEP, together with those from other pivotal PrEP studies [13,14] informed the advice by the Dutch National Health Council to the Ministry of Health to roll out PrEP in the Netherlands. In 2019, subsidised PrEP and related care became available for 8,500 individuals in the Netherlands, including 2,900 in Amsterdam. There are currently no data available on the estimated PrEP need in Amsterdam. 

Individuals with acute HIV infection (AHI), who generally have very high viral loads, have a particularly high likelihood of transmitting HIV [15]. A study on HIV transmission that combined viral phylogenetics and detailed clinical and demographical data, showed that 70% of all forward transmissions in MSM in the Netherlands occurred within 3 months of HIV infection [16]. These insights prompted us to design a specific AHI test-and-immediate-treat pathway by which MSM and TGP can self-refer through an online AHI awareness tool [17] or be referred by their general practitioner or the CSH in Amsterdam. This AHI test-and-treat pathway used a symptom recognition score, adopted to fit the characteristics of individuals with AHI [18], which is obtained through the website’s symptom checker tool, and a point-of-care HIV RNA test. The observation that this AHI test-and-treat pathway significantly decreased the time between AHI diagnosis and viral suppression [19] led to the adaptation of this AHI test-and-treat pathway in routine care at the CSH in Amsterdam in 2019. After implementation of this approach, there was an increase in the proportion of newly diagnosed MSM and TGP in Amsterdam with evidence of recent infection (Figure 2).

One of the remaining challenges is reducing the proportion of individuals diagnosed with late-stage HIV infection which has been around 35% of new diagnoses since 2010. Of the 79 people with a late-stage HIV diagnosis in 2020 to 2022, almost half (n = 37) were diagnosed in a hospital [9]. Several studies demonstrated that, despite European guideline recommendations [20], testing for HIV in the presence of an HIV indicator disease is still not routine practice in hospitals and therefore, opportunities for earlier diagnosis are frequently missed [21]. To improve earlier diagnosis, indicator-based testing interventions were implemented in Amsterdam in 2019 to improve adherence to HIV indicator disease-based testing guidelines in Amsterdam hospitals [22].

## Discussion

The H-TEAM Initiative shows that a city-centred effort [23], addressing multiple aspects across the HIV prevention and care continuum, is a feasible approach and has contributed to an estimated 95% decline in new infections, to the 79% decline in new HIV diagnoses and to Amsterdam reaching the 95–95–95 treatment cascade target in 2022. Despite these milestones, a small number of individuals remain viraemic either because they are not yet diagnosed or are disengaged from HIV care. Unfortunately, we do not have data on the individuals that were not retained in care. A likely explanation is that some of them may have died or moved abroad without notification to the SHM. 

## Conclusion

Currently, H-TEAM stakeholders are addressing the last mile to zero new infections by investigating the efficacy of novel interventions to improve outreach to these individuals. These approaches include tailored HIV and sexual health, PrEP care, insight in PrEP need and generating a more detailed epidemiological and sociological picture of the HIV epidemic at city level, using geographical information systems technology. This information, together with our described successful subprojects on prevention, testing and treatment, will hopefully lead to Amsterdam being one of the first cities worldwide that eliminates new HIV infections in the coming years.

## The HIV Transmission Elimination AMsterdam (H-TEAM) Initiative

Ard van Sighem (Stichting HIV Monitoring, Amsterdam, the Netherlands), Sally Hendriks (Soa Aids Nederland, Amsterdam, the Netherlands), Febe Deug (Soa Aids Nederland, Amsterdam, the Netherlands), Paul Zantkuijl (Soa Aids Nederland, Amsterdam, the Netherlands), Jan EM van Bergen (Department of General Practice, Amsterdam University Medical Centers, University of Amsterdam, the Netherlands), John de Wit (University of Utrecht, Utrecht, the Netherlands) , Janneke Heijne (Department of Infectious Diseases, Public Health Service of Amsterdam, Amsterdam, the Netherlands), Elske Hoornenborg (Department of Infectious Diseases, Public Health Service of Amsterdam, Amsterdam, the Netherlands), Tom van Benthem (Department of Infectious Diseases, Public Health Service of Amsterdam, Amsterdam, the Netherlands), Maarten F Schim van der Loeff (Department of Infectious Diseases, Public Health Service of Amsterdam, Amsterdam, the Netherlands), Maria Prins (Department of Infectious Diseases, Public Health Service of Amsterdam, Amsterdam, the Netherlands), Udi Davidovich (Department of Infectious Diseases, Public Health Service of Amsterdam, Amsterdam, the Netherlands), Suzanne E Geerlings (Department of Internal Medicine, Division of Infectious Diseases, Amsterdam University Medical Centers, University of Amsterdam, and Amsterdam Infection and Immunity Institute, Amsterdam, the Netherlands), Dinah Bons (Trans United Europe), Michiel Heidenrijk (Amsterdam Health Technology Institute, Amsterdam, the Netherlands), Pieter Brokx (Dutch HIV Association, Amsterdam, the Netherlands), Nina Schat (Department of Global Health, Amsterdam University Medical Centers, University of Amsterdam, and Amsterdam Institute for Global Health and Development, Amsterdam, the Netherlands), Peter Reiss (Department of Global Health, Amsterdam University Medical Centers, University of Amsterdam, and Amsterdam Institute for Global Health and Development, Amsterdam, the Netherlands), Marc van der Valk (Stichting HIV Monitoring, Amsterdam, the Netherlands; Department of Internal Medicine, Division of Infectious Diseases, Amsterdam University Medical Centers, University of Amsterdam, and Amsterdam Infection and Immunity Institute, Amsterdam, the Netherlands) Godelieve J de Bree (Department of Internal Medicine, Division of Infectious Diseases, Amsterdam University Medical Centers, University of Amsterdam, and Amsterdam Infection and Immunity Institute, Amsterdam, the Netherlands).
